# Impact of Fitness Status on the Optically Measured Hemodynamic Indexes

**DOI:** 10.1155/2018/1674931

**Published:** 2018-01-09

**Authors:** B. I. Kuznik, Y. N. Smolyakov, S. O. Davydov, N. N. Tsybikov, O. G. Maksimova, A. V. Malinina, L. Shenkman, A. Kaminsky, I. Fine

**Affiliations:** ^1^Chita State Medical Academy, Chita, Russia; ^2^Innovative Clinic “Health Academy”, Chita, Russia; ^3^Elfi-Tech Ltd., 2 Prof. Bergman St., Science Park, Rehovot, Israel

## Abstract

The physiological characteristics of skin blood flow can be described in terms of the hemodynamic indices (HI). The HI is derived from the laser speckle characteristics, which are governed by the cutaneous blood flow. A miniaturized dynamic light-scattering sensor was used to measure the speckle pattern from the finger root. Three groups of subjects from 15 to 25 years of age were tested. The first group included subjects who are actively engaged in sport activities; the second group included subjects with low level of physical activity; and the third group included healthy controls with moderate physical activity. The HI parameters were measured prior to and after the performance of a determined physical load. As a marker of cardiovascular fitness (CVF), we used the postload decay rate of HI. We found that the hemodynamic response to the physical load provides a statistically significant correlation with the postload heart rate decay. It was also found that postocclusion increase of the arterial HI is more prominent in the group with higher physical activity. These results indicate that hemodynamic indices can be used as an additional marker for cardiovascular fitness level.

## 1. Introduction

### 1.1. Noninvasive Markers of Fitness

Reducing illness and promoting wellness and fitness are important goals for the healthcare system. Wellness self-management can be a key factor in improving the quality of life. Like any skill, self-management skills must be practiced if they are to be useful. Therefore, providing feedback regarding the improvement of the key physiological parameters by physical activity and diet is an important factor for such self-management. Cardiovascular fitness (CVF) is assessed by the ability of the heart and respiratory system to withstand a load, as well as by the ability of our sympathetic and parasympathetic system to respond to rapid changes. While addressing the daily home measurement of these parameters, it is apparent that only a seamless device using noninvasive CVF markers will meet the requirements of self-monitoring.

Today, only a few noninvasive markers for the assessment of different aspects of fitness have been adopted. Each marker addresses different aspects of cardiovascular health. The most popular parameter is the heart rate (HR). Resting HR can vary with fitness level and with age. The HR mainly relates to the efficiency of heart to pump the blood through the body. The association between a high level of cardiorespiratory fitness (physical fitness) and a low resting heart rate is well known [1]. Many studies have suggested that physical activity (PA) levels and CVF impact on the autonomic control of heart rate (HR) [2]. During exercise, the sympathetic nervous system dominates the regulation of body functions. Therefore, after exercise, the sympathetic nervous system withdraws and the parasympathetic nervous system helps the body return to a resting state. Training mode showed statistically significant effects on the speed of heart rate recovery in trained subjects.

An additional important noninvasive marker of CVF is heart rate variability (HRV) [3]. HRV is mediated by the autonomic nervous system (ANS) which regulates different functions of the body through the parasympathetic and sympathetic subsystems [3, 4].

Another well-established optical technique is the near-infrared spectroscopy (NIRS), which allows for the determination of tissue and blood analytes based on spectrophotometric measurements in the visible and near-infrared regions of the spectrum of light [5]. According to this technique, incident light penetrates the examined skin, and reflected and/or transmitted light is/are measured. Optical plethysmography, pulse oximetry, and occlusion spectroscopy are the most prominent examples of usage of the NIR spectroscopy in medical and physiological studies. Visible or near-infrared light is commonly used to track the optical manifestation of some time-dependent physiological processes. Such prolonged measurement of light response as a function of time can provide the clinician with valuable information about time-dependent physiological processes. For example, the measured light response of a natural heartbeat pulsation is varied with each pulse. The signal is then measured at one point of the pulse wave and compared with the signal at another point. The difference between the values is due to arterial blood alone. In pulse oximetry, this phenomenon is utilized for the determination of oxyhemoglobin saturation. The major underlying assumption in the processing of all kind of time-dependent signals is that the measured optical variation is originated solely by blood-related components. In pulse oximetry, for example, it is commonly accepted that arterial blood volume changes are the only responsible factors behind the optical signal modulation. However, a more complex physical analysis shows that even if the only changes in the system are ascribed to the blood, the measured optical response of these changes is a convolution of absorption and scattering properties of blood and surrounding media. While carrying out any algorithmic modeling and signal processing procedure of these measured optical signals, the tissue-related effects cannot be disregarded. Therefore, the common denominator of all time-dependent signal-related optical methods relies on the measurement of optical responses originated by the blood dynamics or hemorheological status changes. However, NIR-related techniques provide only indirect information regarding the hemodynamics of the human body.

Each of these parameters mentioned above provides very important information about different aspects of cardiovascular function. However, none of them addresses directly the hemodynamic responses of the body. The hemodynamic response is defined not only by the heart and respiration system but also by vascular resistance. One of the key factors affecting the vascular resistance of blood vessels is the functioning of the endothelial cells. The role of endothelial cells is, therefore, an additional essential characteristic related to cardiovascular health [6]. In order to provide a comprehensive assessment of CVF of the body, it is necessary to determine the interplay between heart performance, ANS adjustability, and vascular functioning.

## 2. Materials and Methods

### 2.1. Miniaturized Dynamic Light-Scattering Sensor

**Figure 1:**
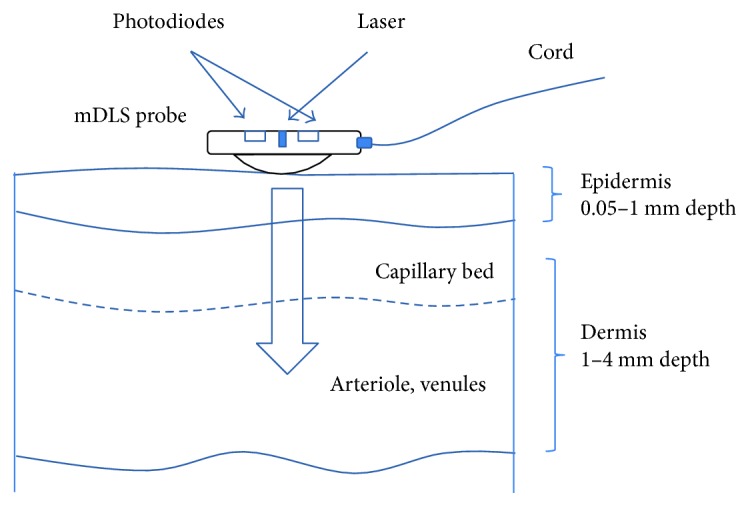
Schematic geometry of the mDLS sensor.

The miniaturized dynamic light-scattering sensor (mDLS) is designed to measure different parameters of the cutaneous blood flow [7]. Laser light is scattered from tissue and moving red blood cells (RBC). The RBC movement gives rise to the intensity fluctuations of the reflected light (see Figure 1). The RBCs are the major contributor into the signal because of a few reasons; first of all, the red blood cells are the most common particles in the blood. There are around 4 million RBCs per microliter of blood. There are only about 4000–11,000 of white blood cells per microliter and about 150,000–400,000 platelets. Moreover, each RBC cell includes the hemoglobin molecules. Thanks to this, the RBCs have a strong mismatch in refractive index and the scattering coefficient of the RBC is almost 1000 times larger than the scattering coefficient for any other blood particle. Combining these facts, we understand the scattering from all the rest of the particles is negligible in comparison to the RBCs.

Thus, the RBC scattered light intensity fluctuations are manifested by the laser speckle appearance on the area of the detector. In the mDLS sensor, because of the very small distance between the laser and photodetectors, most of the light is scattered directly to the detector.

Therefore, the single scattering component prevails and the multiple scattering components are practically negligible. This means that the measured signal can be expressed explicitly in terms of optical properties of the RBCs and their movement characteristics.

The reflected signal is measured by two detectors, which are located in close vicinity to each other. The sampling frequency of the system was 48 kHz. There is no spatial analysis done for these two signals. The purpose of these two detectors is to reject any zero frequency or ambient interferences. By manipulating these two analog electrical signals, it is possible to generate a third or “hybrid” analog electrical signal (e.g., a “difference” electrical signal describing difference between the first and second analog electrical signals). This method enables rejection of all mutually correlative components for two detectors (e.g., ambient light) while the stochastic component survives. Therefore, the area between these two detectors is essentially measured and the intensity fluctuation of the combined signal as a function of time is analyzed.

The second order statistics of the dynamic appearances of the speckles at the spot area located between these two detectors are a subject for further analysis. These statistics can be described in terms of the Doppler shift from the moving RBCs. Alternately, the same phenomena can be analyzed by considering the interferences of the scattered light on the detector surface. The last model resembles the dynamic light-scattering (DLS) approach. These two formalisms essentially provide the same results and describe the relationship between the movement of the RBCs and the time fluctuation of the measured light intensity.

### 2.2. The Measurement Device

The measuring device Elfor3 (Elfi-Tech Ltd.) consists of the following subunits: the sensor probe, the pressurizing module, and the acquisition system.

**Figure 2:**
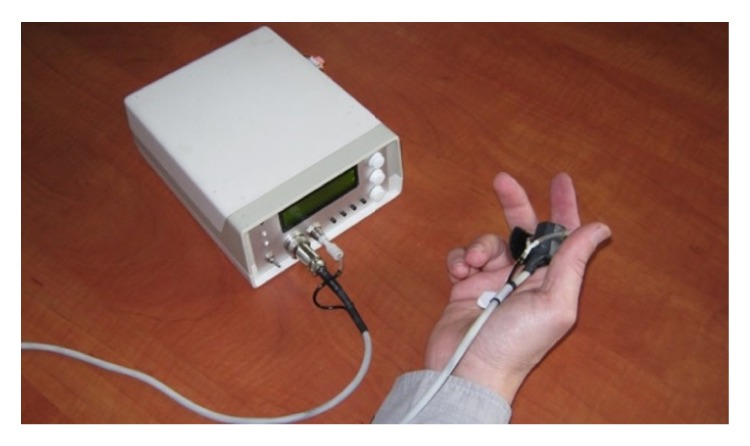
Device application.

The sensor probe is incorporated in a mechanical enclosure, which can be fixed on the finger (Figure 2). In addition, it includes the pneumatic cuff. The air pressure in this cuff is controlled by the device. Following the control command, over systolic pressure (above 250 mmHg) can be automatically applied. Thus, the cuff provides local compression of the arterioles, resulting in the cessation of blood flow under the area of measurement. In the current study, the predetermined measurement mode was used to create pre- and postocclusion measurement stages.

The hemodynamic vascular response to the occlusion event may be used to evaluate the hyperemia response, which is one of the characteristics of endothelial functioning.

### 2.3. Description in terms of Blood Flow Velocity Gradient

The classic model of laser speckle analysis for randomly moving particles can be adopted for the description of the signals related to the blood flow. In the standard DLS model, the relative movement of the scatterers is expressed through the diffusion coefficient. This description can be extended for the laminar blood flow. According to this model, the laser speckle dynamics are governed by the distance changes between the closely located moving RBC. The rate of this change is defined by the relative velocity of the moving RBCs [8]. The well-known term “shear rate” is one of the characteristics of the velocity gradient. The very existence of this gradient in flowing blood gives rise to the laser speckle dynamics.

For laminar flow, the velocity gradient is defined by

(1) $$\begin{array}{c} \nabla v\left(r\right)=v\left(x,r\right)-v\left(x,r+\nabla r\right). \end{array}$$

*v*(*x*, *r*) and *v*(*x*, *r* + ∇*r*) are representing the RBC velocities in two adjacent layers of flow. In a very simplified case, for the vessel of radius *R*, axis symmetric velocity profiles *v*(*r*, *t*) can be described in cylindrical coordinates by this empirical relationship:

(2) $$\begin{array}{c} v\left(r,t\right)=v\left(0\right)\cdot \left[1-{\left(\frac{r}{R}\right)}^{\xi }\right]\cdot f\left(t\right), \end{array}$$

where *v*(0) is the maximum velocity at the center position *r* = 0 and *R* is the radius of the vessel, *f*(*t*) is a periodic function of heartbeat frequency, which is driven by difference between systolic and diastolic pressure wave and it is time phase-shifted with respect to the cardiac cycle. *ξ* represents the degree of blunting. Degree of blunting *ξ* ranges from 2 for a parabola to 1 for complete plug flow.

By differentiating (1), we determine that

(3) $$\begin{array}{c} \nabla \left[v\left(r\right)\right]=\frac{v\left(0\right)\cdot \xi \cdot {\left(1-\left(r/R\right)\right)}^{\xi -1}}{R}. \end{array}$$

From (3), it is seen that the velocity gradient is determined both by the diameter of the vessels and by the distance of the moving RBCs from the vessel's wall.

For very small arterioles (from 15 to 60-micron diameter), the average velocity is around 10 mm/sec, while for the capillaries ranging from 5 to 10 microns, the average velocity is around 0.2 mm/sec. From (3), it is obvious that the maximum values of the gradient are achieved around the central part of the vessels. The gradient gradually drops closer to the vessel walls. Practically, the near-wall velocity never reaches zero values, as it apparently appears in (3), since active functioning of endothelial cells prevents it.

The main goal of the presented study was to evaluate the use of laser speckle technique to create new noninvasive markers for CVF by measuring hemodynamic characteristics derived from the measured signal.

### 2.4. Dynamic Light Scattering and Shear Rate

In DLS theory, one of the most important characteristics of the intensity fluctuation is the autocorrelation function of this signal *g*(*τ*). Approximately, in laminar blood flow, the characteristic decay time of autocorrelation function can be given by [8]

(4) $$\begin{array}{c} \overset{.}{g\left(\tau \right)\propto \exp\left(-\Gamma \tau^{2}\right)}, \end{array}$$

where Γ is dependent on the velocity gradient, wavelength, and measurement geometry.

Practically, the power spectrum of the DLS represents a convolution of all components of the blood velocity gradients [9]. A proper analysis of the power spectrum characteristics can reveal the fine features of the cutaneous blood flow.

By using the power spectrum (*P*) presentation of (4), the hemodynamic index (HI) can be defined by [7, 10]:

(5) $$\begin{array}{c} HI\left(\left[f_{1},f_{2}\right],t\right)=\int_{f_{1}}^{f_{2}}P\left(\omega ,t\right)d\omega , \end{array}$$

where *f*_1_ and *f*_2_ are defined as chosen bandpass bounders.

Physiologically, the interpretation of different HIs is related to different velocity gradients, as defined by (3). High-frequency HI(*t*) exhibits a pulsatile pattern and therefore is more associated with the blood flow in the arterioles. The nonpulsatile HIs are associated with the capillary blood and near the vascular wall layers of blood.

Below are examples of 3 types of HI. HI_1_ corresponds to the very low velocity gradient, presumably representing the near vascular walls and very small capillary blood, HI_2_, low velocity gradient, represents mainly the capillary blood, and HI_3_, high-frequency component, corresponds to pulsatile movement of the RBCs.

**Figure 3:**
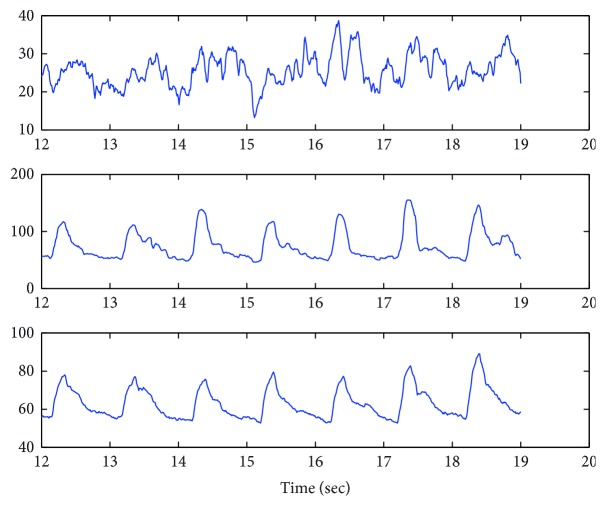
HIs represent different blood flow components. The first graph is HI_1_, the second is HI_2_, and the third is HI_3_.

**Figure 4:**
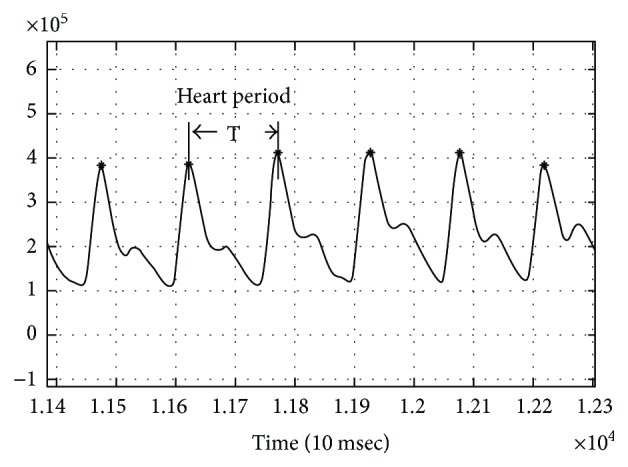
Waveform of the signal and intervals between the peaks.

As seen in Figure 3 and Figure 4, the pulsatile component is very similar to the classic PPG signal, while the two other components lack any PPG-like appearance of the pulsatile waveform.

Of note, the HI value is also proportional to the number of scatterers. In order to address only the shear rate characteristics, we introduced the normalized or relative hemodynamic index (RHI_*i*_) defined by the following:

(6) $$\begin{array}{c} {RHI}_{i}=\frac{{HI}_{i}}{\int_{0}^{f_{end}}P\left(\omega ,t\right)d\omega }, \end{array}$$

where *f*_end_ is Nyquist value of the measurement sampling frequency. In our specific case, *f*_end_ = 8 kHz.

RHI signifies relative contribution of different blood components, like endothelial, capillary, or arterial into DLS signal.

### 2.5. Study Protocol

In our study, the mDLS sensor was attached to the palmar side of the finger. HI matrix formed by three equidistant (in log scale) frequency bands: HI_1_ ranging up to 220 Hz, HI_2_ to 221–1324 Hz, and HI_3_ to 1325–8000 Hz. HI_1_ is associated with the RBCs with very low velocity gradients, HI_2_ index represents mainly capillary blood, and HI_3_ characterizes arterial stream of blood.

The pilot study involved 120 people of both sexes aged 21 ± 4 years. The subjects were divided into three groups, according to the reported physical activity level: 19 subjects were physically nonactive, 92 subjects engaged in moderate physical activity, and 19 subjects declared that they actively participate in training and sports.

The study protocol included the following activity stages: (a) quiet sitting measurement, (b) dosed physical activity in the form of 10 squats, and (c) quiet sitting after the exercise.

All measurements prior to the physical load are defined as a baseline (“CONTR”). All measurements after the physical load are defined as “STRESS.” During control sessions, the measurement protocol included recording mDLS signal for 10 seconds without occlusion (T1), 20 seconds when the cuff is inflated to 200 mmHg (T2), and recording mDLS signal for 10 seconds after deflating the cuff (T3).

During “STRESS” or postexercise session, all measurements lasted 60 seconds, where the first 10 sec (T1) was used for the determination of initial HR (T1), HI_*i*_ (T1), and RHI_*i*_ (T1) and the last 10 sec (T2) was used for the determination of final HR (T2), HI_*i*_ (T2), and RHI_*i*_ (T2).

The matrix of hemodynamic indices (HI_*i*_) and RHI_*i*_ was calculated for each of the bandpasses for each time slot after exercise for “CONTR” and for “STRESS” sessions.

In addition, the pulse rate HR in beat/min during each session was determined. Delta HR is defined as the difference between the HR at the first 10 seconds immediately after the physical activity (T1) and by the end of 1 min (T2).

## 3. Results and Discussion

### 3.1. Postphysical Activity Responses as Function of Pulse Rate Changes

For every subject, we calculated the heart rate by using the waveform of the signal during the pre- or postocclusion session. The waveform was obtained by using HI_3_, decimating to 20 Hz, and by applying the bandpass filter between 0.4 and 3 Hz.

Following these steps, the systolic peaks were identified. The distance between the peaks was assumed as an approximation of beat-to-beat interval. By averaging this parameter for all peaks during the pulsatile session of the signal, the heart rate (HR) can be easily determined. It has to be pointed out that the information for the pulse was derived from the HI_3_ while further analysis was performed by juxtaposing HI_2_ and HR. Therefore, no hidden correlation exists between the nonpulse perfusion related to HI_2_ and pulse rate derived from the waveform of HI_2_.

For every subject, we defined *α* = Δ(HR)/HR where Δ(HR) is the difference between the HR immediately after the load and the HR during the rest stage. This parameter reflected a CVF by the postload relaxation characteristics. The faster the recovery rate and the lower the HR, the higher is *α*.

**Figure 5:**
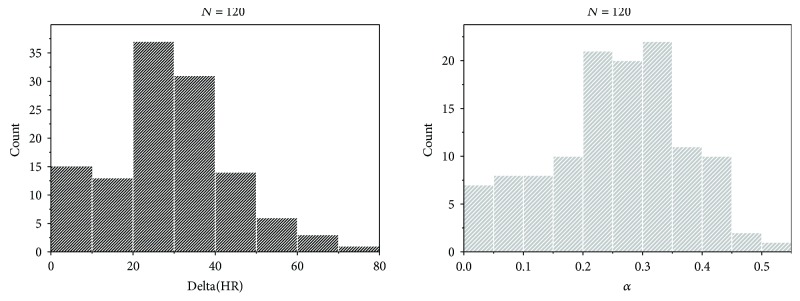
Distribution of the Δ(HR) and *α* over the subjects.

**Figure 6:**
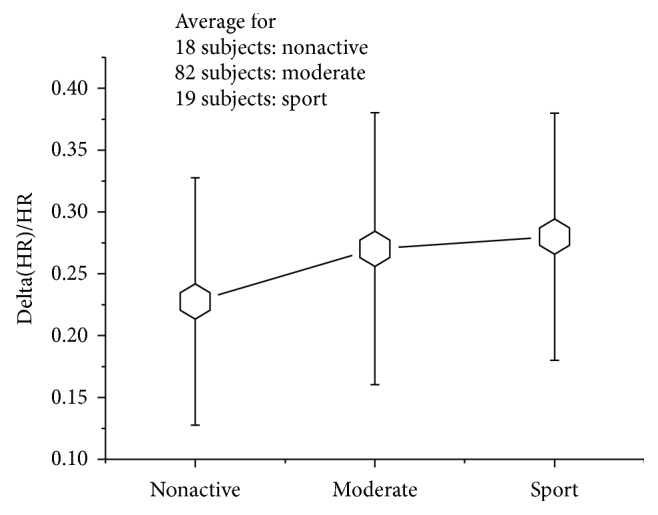
Mean value of *α* for three groups of the subjects.

Figure 5 shows distribution of the ΔHR and *α* values for all tested subjects:

In the following graph, we can see the behavior of the average value of *α* for three groups of subjects: nonactive, moderate, and sport:

From Figure 6, we can see that *α* tends to increase as a function of physical activity. This result is consistent with the expected correspondence between *α* and CFV. In our further analysis, we used *α* as an objective reference that one can rely upon for investigating the assumption that there is a correlation between the hemodynamic indexes and CVF.

The most prominent correlation between the hemodynamic indexes and *α* was found for the HI_2_ and RHI_2_ taken at the initial stage after the exercise or relaxation onset.

**Figure 7:**
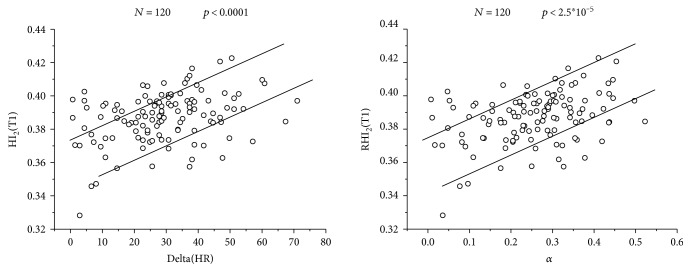
Correspondence between (RHI_2_ (T1)) and ΔHR and between RHI_2_ (T1) and *α*.

In the next graph (Figure 7), the correspondence between the HI_2_ (T1) and RHI_2_ (T1) and *α* is presented:

As seen in the statistical analysis, there is a very significant correspondence (*p* < 10^−5^) between the RHI_2_ (T1) with the relative change of the heart rate (*α*) and the delta (HR) (*p* < 0.0001). This result indicates that capillary hemodynamic response at the initial stage of postexercise recovery is strongly related to the CVF.

### 3.2. Hyperemic Response to Occlusion

**Figure 8:**
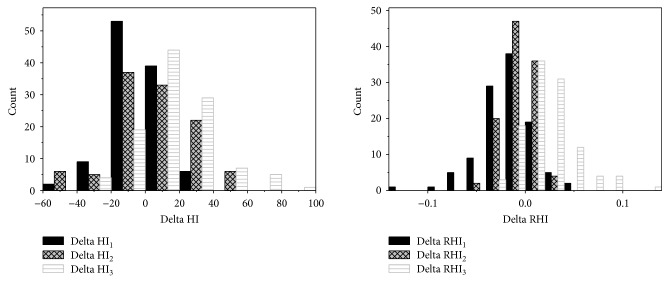
Hyperemia response for three hemodynamic indexes.

In order to evaluate the endothelial functioning, we measured the postocclusion hemodynamic index responses.  Figure 8 shows that HI_3_ and RHI_3_ tend to increase after the occlusion while HI_1_ and RHI_1_ indexes mostly decrease.

**Figure 9:**
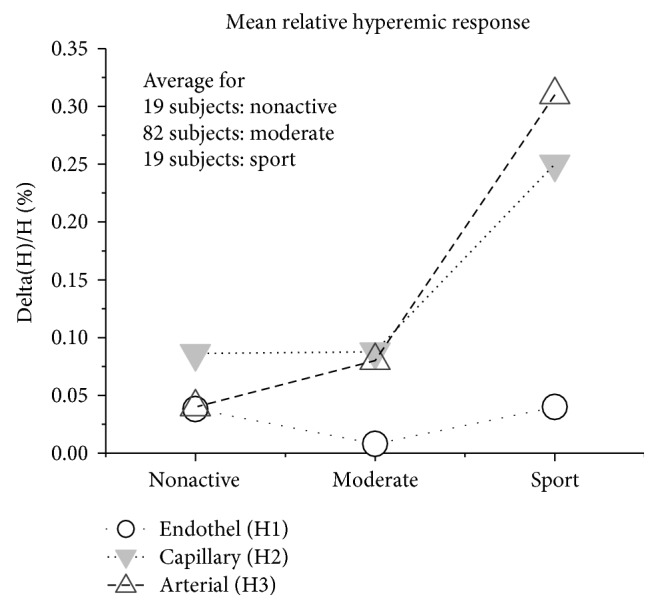
Total perfusion at sessions T1 and T3 versus RHI_2_.

Finally, we compared a strength of hyperemic response for the three groups of subjects: nonactive, moderately active, and active-sort group.  Figure 9 shows that the most significant responses are associated with the subjects who are engaged in sport activity.

The results of the different response to the occlusion stimulus can be again explained assuming that there is a difference in endothelial functioning between healthy/sport subjects and the nonactive group. Of note, Vane et al. [6] found a correlation between coronary and peripheral microvascular endothelial functioning. In [11], it was shown that digital reactive hyperemic response differentiates between healthy subjects and persons with atherosclerosis. It means that functioning of endothelial cells significantly affects the hyperemia response in the finger. Since HI_3_ is associated with arterial blood flow, it is not surprising that more fit individuals and athletes exhibit better endothelial response to hyperemia than other groups.

## 4. Conclusions

We have shown a statistically significant correlation between the hemodynamic indexes derived at the onset of the postload session and the heart rate relaxation rate, which is a widely recognized marker of CFV. In addition, we demonstrated that the typical reactive hyperemia response, when expressed in terms of hemodynamic indexes, enables the differentiation between athletes and other less trained groups. This result indicates that HI parameters can be used for an assessment of endothelial functioning.

All these findings taken together provide solid evidence that hemodynamic indexes can be used as a new noninvasive marker for CV and endothelial performance of the human body.
